# Case report: A case of giant malignant solitary fibrous tumor of the pleura with Doege-Potter’s syndrome and review of the literature

**DOI:** 10.3389/fonc.2024.1437535

**Published:** 2024-11-29

**Authors:** Jie Li, Hong-Tao Tang, Qing Liu, Cai-Han Li, Wei-Yang Chen, Zeng-Wei Yu, Lei Wang, Lin Lin, Jin-Lan Zhao, Chun-Yan Zhao, Long-Qi Chen, Dong Tian

**Affiliations:** ^1^ Department of Thoracic Surgery, West China Hospital, Sichuan University, Chengdu, China; ^2^ Department of Cardiovascular Surgery, West China Hospital, Sichuan University, Chengdu, China; ^3^ Integrated Care Management Center, West China Hospital, Sichuan University, Chengdu, China; ^4^ Department of Radiology, Key Laboratory of Birth Defects and Related Diseases of Women and Children of Ministry of Education, West China Second University Hospital, Sichuan University, Chengdu, China; ^5^ Department of Thoracic Surgery, The First Hospital of China Medical University, China Medical University, Shenyang, China; ^6^ Anesthesia Surgery Center, West China Hospital, Sichuan University/West China School of Nursing, Shenyang, China; ^7^ Department of Nuclear Medicine, West China Hospital, Sichuan University, Chengdu, China

**Keywords:** solitary fibrous tumor of the pleura, Doege-Potter’s syndrome, surgical resection, surgical approach, malignant lesion, hypoglycemia

## Abstract

The solitary fibrous tumor of the pleura (SFTP) is a rare intrathoracic neoplasm that commonly originates from the subpleural mesenchymal cells of the visceral pleura and accounts for less than 5% of all pleural tumors. We reported a case of a 54-year-old man with a two-week history of hypoglycemia, a six-month history of productive cough and fatigue, and chronic right chest pain. Radiological techniques revealed a giant intra-thoracic mass with hypervascularization, and pathological staining was carried out to make a definitive diagnosis of SFTP. Interventional embolization was conducted to block the main feeding vessels before the surgery, and an anterolateral thoracotomy combined with a transverse sternotomy was performed to achieve a complete resection, which demonstrates significant potential for further application in patients with unilateral giant SFTP. The postoperative course was uneventful, with no signs of hypoglycemia observed during the follow-up. Additionally, we reviewed and prospected the research progress on SFTP. The aim of this study is to enhance clinicians’ understanding of SFTP through our case and to provide a detailed review of the current research.

## 1 Introduction

Solitary fibrous tumor of the pleura (SFTP) is a rare neoplasm that originates from the subpleural mesenchymal cells (1, 2). It often arises from the visceral pleura and comprises fewer than 5% of all pleural tumors, with an incidence of approximately 2.8 cases per 100,000 registered hospital patients. The incidence of SFTP peaks at 60-70 years old, is infrequent in childhood and shows no apparent gender predilection (3–7). In general, SFTP grows slowly and remains asymptomatic until incidentally discovered during a physical examination. Consequently, a small neoplasm can continuously enlarge into a giant mass, causing significant compression symptoms and, in a small number of patients, is accompanied by paraneoplastic syndromes, such as Doege-Potter’s syndrome (hypoglycemia) and Pierre-Marie-Bamberg syndrome (arthritis) (8). Herein, we present a rare case of a giant malignant SFTP with Doege-Potter’s syndrome and a literature review of current research.

## 2 Case description

A 54-year-old previously healthy man was admitted to our tertiary medical center for further evaluation of a two-week history of hypoglycemia-induced dizziness, a six-month history of productive cough and fatigue, and chronic right chest pain. Two weeks prior, he had been hospitalized at a local secondary care hospital due to severe nocturnal hypoglycemia (with blood glucose levels around 1.1 mmol/L) and was found to have a large abnormal opacity on the chest X-ray. Only conservative treatments were administered before he was transferred to our hospital.

**Figure 1:**
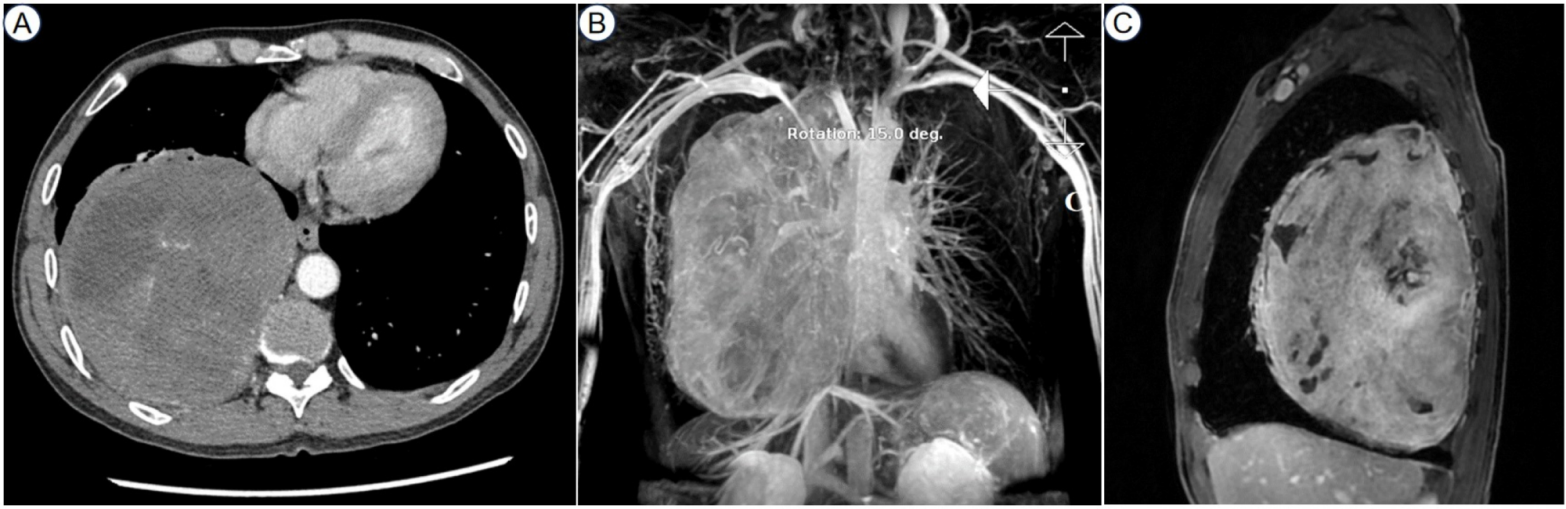
The contrast-enhanced chest CT showed a giant intra-thoracic heterogeneous mass occupying the right chest cavity **(A)**. The CT angiography reconstruction revealed hypervascularization **(B)**. The contrast-enhanced MRI presented uneven enhancement within the tumor **(C)**. CT, computed tomography; MRI, magnetic resonance imaging.

**Figure 2:**
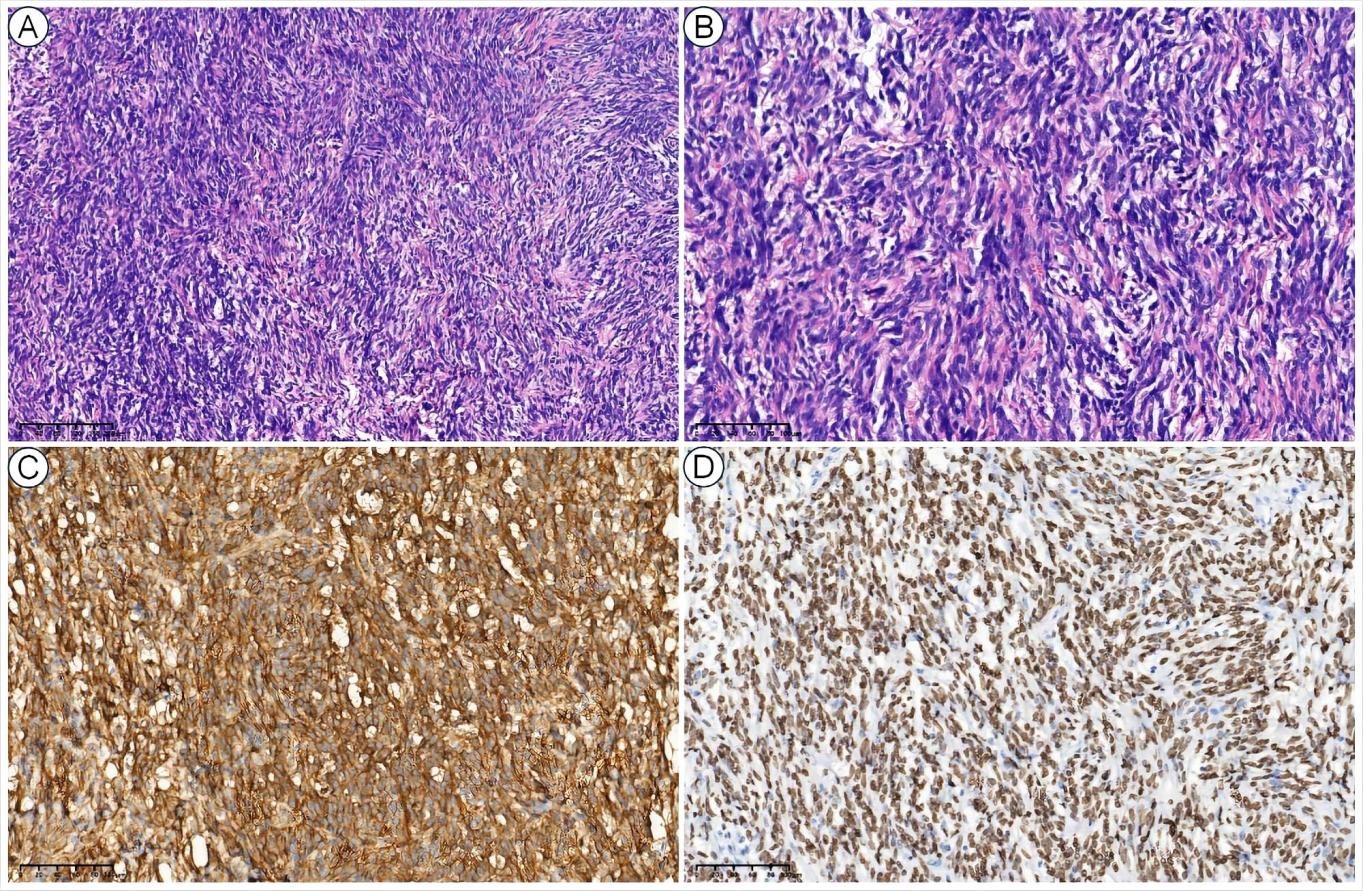
Histopathological views of SFTP. The low-power (10×) hematoxylin and eosin (H&E) view showed a patternless pattern **(A)**, and the high-power (40×) H&E view showed enlarged spindle cells with a variable proportion of collagenous stroma **(B)**. The tumor cells were also positive for CD34 **(C)** and STAT6 **(D)**.

Comprehensive work-ups were performed following his admission to our hospital. The pulmonary function tests indicated severe mixed ventilatory defects. The contrast-enhanced chest computed tomography (CT), CT angiography reconstruction, and contrast-enhanced magnetic resonance imaging (MRI) revealed a giant intra-thoracic heterogeneous mass occupying the right hemithorax, resulting in a complete collapse of the right lung. Hypervascularization was also observed, suggesting a high possibility of malignancy (**Figure 1**). A diagnosis of malignant SFTP was confirmed via percutaneous transthoracic needle biopsy, revealing a patternless pattern characterized by enlarged spindle cells with a variable proportion of collagenous stroma. Tumor cells were positive for CD34, signal transducer and activator of transcription 6 (STAT6), CD99 and B-cell lymphoma 2 (Bcl-2) but negative for smooth muscle actin (SMA), desmin and S100 (**Figure 2**). Meanwhile, the patient received adequate glycemic control and other conservative treatments for pain relief, improvement of lung function, and electrolyte supplementation.

**Figure 3:**
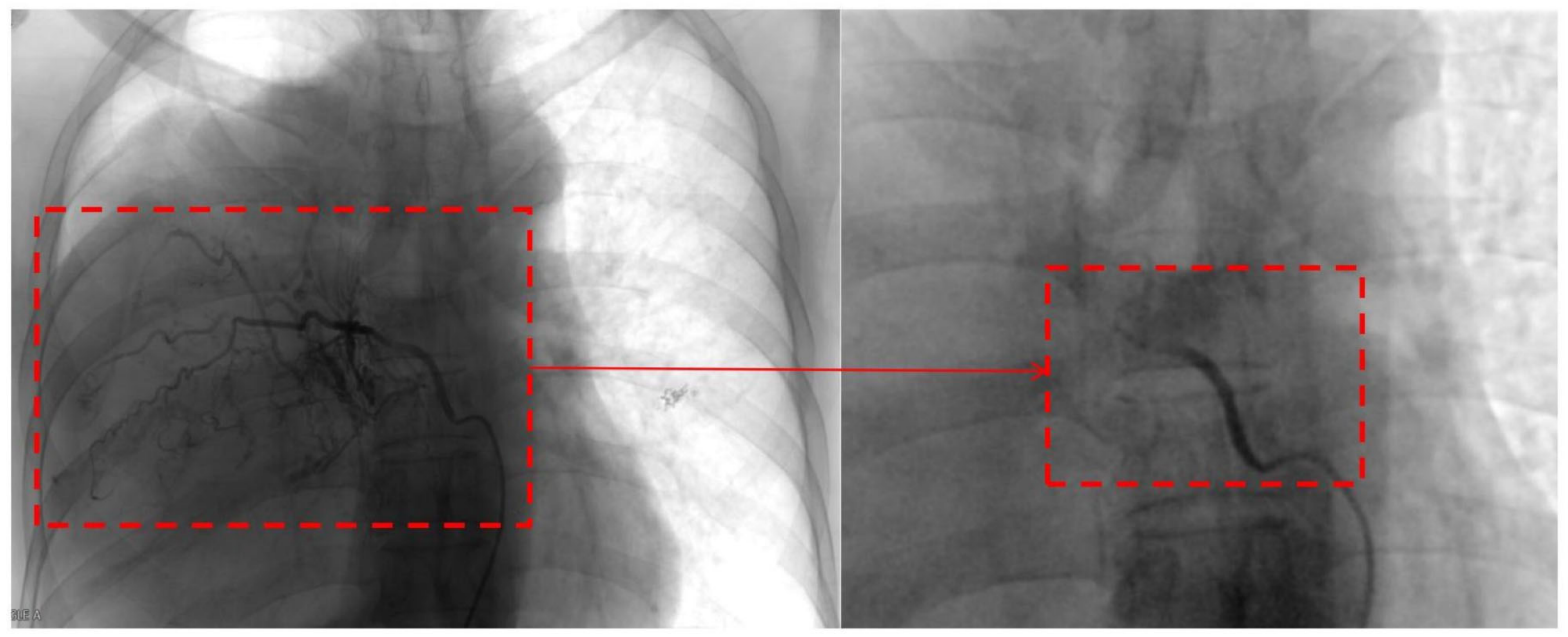
Pre- and post-embolization images of a feeding vessel originating from the bronchial artery.

**Figure 4:**
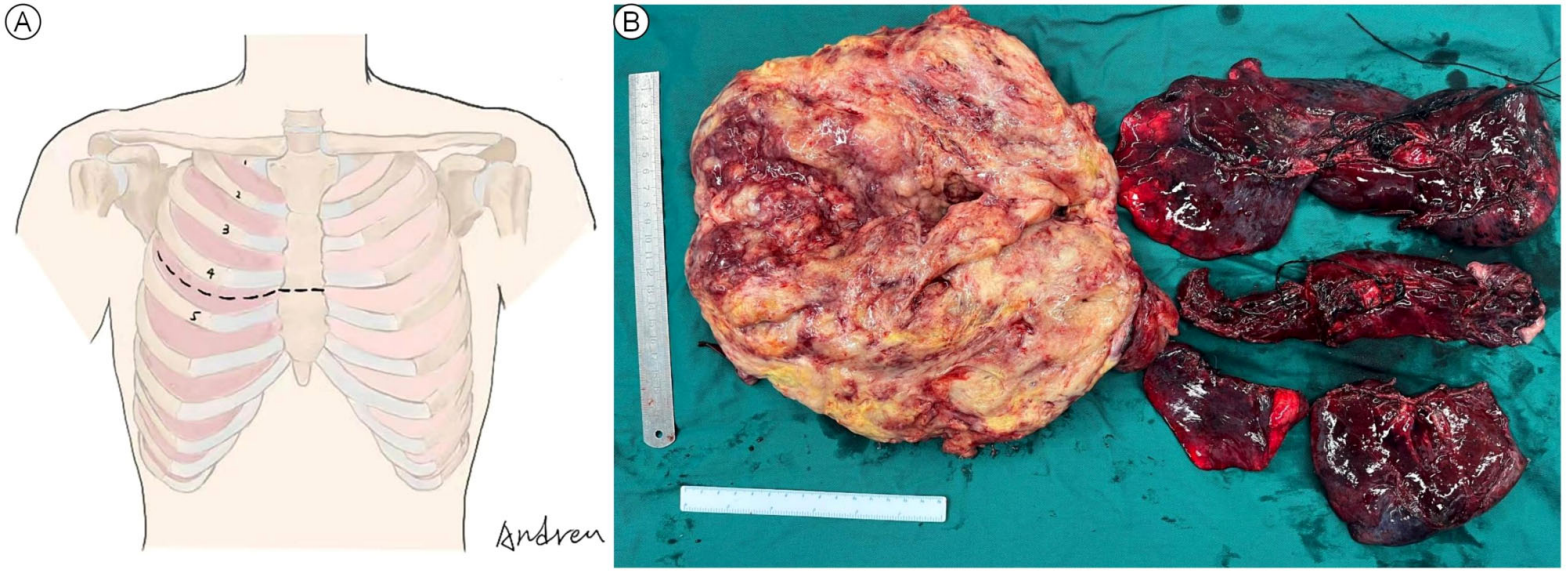
Anterolateral thoracotomy combined with transverse sternotomy **(A)** and the macroscopic view of the resected giant tumor and right lung **(B)**.

The decision of radical resection was made after conservative treatment proved ineffective and the diagnosis of malignant SFTP was well-established. Considering the high risk of hemorrhage, percutaneous angiography via the right femoral artery was performed to confirm the main feeding vessels one day prior to the surgery. Finally, seven feeding vessels originating from intercostal (n = 4) and bronchial arteries (n = 3) were identified, and interventional embolization was conducted using 560-710 μm polyvinyl alcohol particles (**Figure 3**). Due to the large tumor size, a right anterolateral thoracotomy was performed at the fourth intercostal space level, the middle-upper part of the chest, according to the radiological findings, and the sternum was transected to further expand the working space (**Figure 4A**). The giant tumor was then successfully removed, and a right pneumonectomy was also performed, as the right lung had been extensively infiltrated by tumor cells and was no longer functional. Macroscopically, the resected tumor was a yellow-white mass originating from the right posterior parietal pleura, measuring 30 × 20 × 15 cm in size and weighing 5.5 kg (**Figure 4B**). During the operation, the tumor was found closely adherent to the right posterior chest wall, and persistent bleeding may occur if we dissociated the tumor with surgical instruments. The tumor completely filled the right hemithorax, thereby obstructing the visibility of both the tumor’s anatomy and the posterior chest wall. Consequently, only manual blunt dissection was used, and the adjacent chest wall was removed to ensure a tumor-free margin.

The patient exhibited a favorable postoperative recovery, leading to discharge from the hospital three weeks following the surgical procedure. To date, the patient remains alive with no evidence of tumor recurrence or hypoglycemic symptoms. The perioperative blood glucose level and the diagnostic and treatment timeline were presented in the **Supplementary Material** (**Supplementary Figure 1**).

## 3 Discussion

Despite explorations of its epidemiology, pathogenesis, and other aspects in detail, continuous review of SFTP research progress remains essential, given its rarity in clinical practice. Here, we have reviewed and prospected the research progress of clinical manifestations, diagnosis, benign and malignant identification, treatment, and prognosis of SFTP. The key information in this part is summarized in **Supplementary Table 1** (Refer to the **Supplementary Material** for details).

### 3.1 Clinical manifestation

SFTP often manifests with a range of nonspecific respiratory and systemic symptoms, including chest pain, cough, breathlessness, weakness, and weight loss (3, 9). Indeed, most patients, excluding those with large or malignant SFTP, typically remain asymptomatic (10). Doege-Potter’s syndrome and Pierre-Marie-Bamberg syndrome are two uncommon paraneoplastic syndromes among SFTP patients. Doege-Potter’s syndrome is characterized by abnormal insulin-like growth factor-II (IGF-II) produced by tumor cells and occurs in only 5% of patients (8). The abnormal IGF-II produced by tumor cells possesses a high molecular mass and low affinity for other agents, which hinders the formation of ternary complexes. Consequently, it circulates as a binary complex or in the free form, causing hypoglycemia (11). Pierre-Marie-Bamberg syndrome, marked by clubbing fingers, long bone periostitis, and arthritis, is potentially attributed to the excessive release of hyaluronic acid by tumor cells (12). In our case, the patient exhibited refractory nocturnal hypoglycemia along with nonspecific manifestations, such as right chest pain, cough, expectoration and fatigue. These symptoms were rapidly alleviated post-operation, and the blood glucose levels gradually returned to normal. No signs of hypoglycemia were observed during the follow-up.

### 3.2 Diagnosis

Radiological techniques are nonspecific but serve as crucial auxiliary examinations of SFTP (13). Chest X-ray is an economical and widely used technique for thoracic diseases, where SFTP typically manifests as a peripheral opacity with a well-defined margin and homogeneous density (14, 15). CT is valuable in the diagnosis of SFTP, with smaller lesions usually presenting as well-delineated and round or lobulated isodense soft tissue. Larger tumors, however, exhibit internal heterogeneity that correlates with necrosis, hemorrhage or myxoid degeneration on enhanced CT (10, 16, 17). MRI is particularly effective in evaluating the association between tumors and adjacent tissues, where SFTP usually shows low or moderate signal intensity on T1- and T2-weighted imaging, and occasionally, high signal intensity on T2-weighted images can also be observed (17, 18). Although ^18^F-fluorodeoxyglucose (^18^F-FDG) positron emission tomography (PET) or PET/CT imaging can reveal abnormal uptake in SFTP, these techniques are generally not recommended unless metastatic involvement is highly suspected (19). Other radiotracers on SFTP, such as ^68^Ga, have also been investigated to diagnose SFTP (19, 20). While the findings appear promising, additional studies with a large sample size are needed to validate its diagnostic value.

Histopathological findings are pivotal for the final diagnosis. Microscopically, benign SFTP is generally characterized by predominantly spindle tumor cells with abundant collagen deposition, while the malignant one exhibits fewer collagen bundles, presents hemorrhage, varying degrees of fibrosis, and a predominance of spindle cells with pleomorphic, hyperchromatic and anaplastic nuclei (2, 14, 21). Strongly immunopositive CD34 and STAT6 are the most critical diagnostic evidence (9, 22–24). Other immunohistochemical characteristics, such as CD99, Bcl-2, vimentin and Ki-67, can also further support the diagnosis of SFTP. Conversely, SFTP is typically immunonegative for S-100, keratin, SMA, epithelial membrane antigen (EMA), cyclin-dependent kinase 4 (CDK4), mouse double minute 2 homolog (MDM2), and carcinoembryonic antigen (CEA) (13, 14, 21). Importantly, given the heterogeneous and hypervascular nature of SFTP, percutaneous transthoracic needle biopsy may yield false positives, and hemorrhage may also occur once the feeding vessels are impaired.

The importance of genetic diagnosis has earned increasing attention since the discovery of NAB2-STAT6 fusion, which induces cell proliferation and activates early growth response factors, ultimately leading to SFTP (25, 26). This fusion can be easily observed by immunohistochemical staining of STAT6, offering significant diagnostic value (23, 24, 27). Other molecular studies have demonstrated a correlation between p53 mutations, telomerase reverse transcriptase (TERT) promoter mutations, BBS9-BRAF fusion and SFTP, though further validations in larger samples are needed (28–30).

In the present case, radiological techniques revealed a giant heterogeneous tumor in the right thoracic cavity, indicating a high likelihood of malignancy due to hypervascularization, and the definitive diagnosis of malignant SFTP was established through percutaneous transthoracic needle biopsy, underscoring the importance of multidisciplinary diagnosis (**Figures 1**, **2**).

### 3.3 Benign-malignant differentiation

Although most SFTP cases present benign characteristics, approximately 10% to 20% of SFTPs are confirmed to be malignant (2). Historically, the criteria proposed by England and colleagues (31) have been widely accepted for evaluating the malignancy of SFTP. However, the heterogeneity of tumor cells and the subjectivity of pathologists pose challenges to the actual performance of these criteria (1, 5). Efforts have been made to address these deficiencies by incorporating additional objective parameters to create a novel system, achieving comparable or improved diagnostic performance for developing personalized treatment strategies and predicting malignancy and recurrence compared to the criteria established by England et al. (32, 33). Gene markers, such as p53 mutations and BBS9-BRAF fusion, and CT or MRI features that reveal infiltrating growth, hemorrhages, necrosis, and metastasis, also demonstrate the potential to reflect the malignant nature (13, 28, 34). Uneven metabolic patterns or significantly higher uptake within tumors, as depicted by ^18^F-FDG PET or PET/CT scans, are considered helpful by some researchers in distinguishing between benign and malignant SFTP. However, the limited number of studies reporting this effect continues to generate debate (35–38). Notably, there was no positive correlation between tumor size and malignancy, but SFTP with a tumor size greater than 10 cm was reported more likely to be malignant (5, 31, 39). Furthermore, there is no consensus on the definition of a “large” or “giant” SFTP. Current standards appear to depend on the subjective feelings of the researchers (2, 3, 39, 40). In the reported case, although the radiological imaging indicated malignant features, the malignancy of the tumor was ultimately confirmed through needle biopsy. Moreover, the mass we removed was a genuine “giant” malignant SFTP, measuring 30 × 20 × 15 cm and weighing 5.5 kg. To our knowledge, primary malignant SFTP of this size is exceedingly rare. Recently, machine learning methods have achieved significant progress in predicting the biological behavior of tumors, as demonstrated in our previous studies (41, 42), which may also offer a promising breakthrough in identifying malignant SFTP.

### 3.4 Treatment and prognosis

Complete resection remains the mainstay treatment for SFTP (1, 10, 39). Initially, thoracotomy was recommended for patients with SFTP and continues to be the most effective approach to remove the giant one, especially those highly suspected to be malignant (38). With the advent of VATS, small pedunculated tumors are well-suited for VATS with milder postoperative pain and fewer complications (4, 43). For larger pedunculated lesions, combining VATS with mini-thoracotomy can also ensure radical excision (4). Notably, given reports of tumor dissemination through the port in SFTP cases (44, 45), using a wound protector and encasing the tumor in a specimen bag before extracting it through the port when performing VATS is recommended. In the present case, the giant tumor occupied nearly the entire right thoracic cavity, seriously limiting the application of minimally invasive techniques. Thus, a right anterolateral thoracotomy was placed at the level of the fourth intercostal space, and the sternum was transected to further expand the working space, which allowed us to avoid cutting off the ribs, guarantee excellent exposure and facilitate a safe surgical procedure leading to complete en bloc resection. This approach demonstrates immense potential for further application in cases with unilateral giant SFTP and, to our knowledge, has never been applied in SFTP cases.

Giant SFTP often results in severe impairment of lung function due to the mass effect. Therefore, it is imperative to quickly alleviate the compression and preserve as much normal lung tissue as possible. For SFTP originating from the visceral pleura and exhibiting a pedunculated form, wedge resection can ensure radical excision with negative margins. Sessile tumors may require extensive parenchymal resection, which, for larger or giant masses, may include wedge resection or even pneumonectomy (38, 46). For SFTP originating from the parietal pleura, achieving complete resection is more challenging due to typically larger tumor size, wide bases, infiltration of the lung parenchyma or dense adhesion to the chest wall (1). Generally, if the collapsed lung is not infiltrated by tumor cells, it may gradually re-expand once the tumor is excised. However, resection of the infiltrated tissue is recommended to ensure negative margins (15). Additionally, lung tissue may gradually become fibrotic if it remains chronically collapsed due to the mass effect, and surgical removal should be performed to prevent further complications. Notably, since malignant SFTP always presents a high risk of recurrence, a safe margin of 1-2 cm is recommended. A frozen section can be helpful to confirm that the margins are free of tumors (32). In our case, the patient’s right lung, extensively infiltrated by tumor cells, was no longer functional. Thus, a right pneumonectomy was performed, and there was no significant limitation in postoperative respiratory function. Concerning the origin of the tumor, partial resection of the chest wall was added in case of neoplastic invasion of the parietal pleura. Moreover, the hypervascular nature of SFTP should not be ignored. Approximately 50% of SFTP has vascular pedicles that originate from intercostal, mammary, or bronchial arteries (47), and the accidental injury of these feeding vessels may cause unmanageable hemorrhage, even leading to patient death (48). In the reported case, a total of seven feeding vessels were identified through percutaneous angiography and subsequently blocked by interventional embolization using polyvinyl alcohol particles before surgery, significantly reducing intraoperative bleeding. While this preoperative procedure has been rarely reported in previous studies (49, 50), it demonstrates outstanding effects in preventing severe hemorrhage during the operation of a giant SFTP.

Patients with benign pedunculated SFTP generally have favorable prognoses following complete resection, with a 10-year overall survival rate of 97.5% (16). However, local recurrence or metastasis in malignant SFTP cases results in a poor 5-year survival rate of merely 68%, with intrathoracic recurrence proving the most fatal (10). The primary factors contributing to recurrence are improper follow-up and positive surgical margins. Surgical resection remains the most crucial option for managing tumor recurrence and may achieve long-term survival. Palliative radiotherapy and chemotherapy may be considered for patients who are ineligible for surgical resection or temporarily unable to undergo surgery due to excessive tumor size, although the efficacy of these non-surgical treatments lacks consensus (5, 10, 51).

## 4 Conclusion

In conclusion, we presented a rare case of a giant SFTP with recurrent hypoglycemia as a paraneoplastic syndrome and outlined our diagnostic and treatment strategies. We also conducted a comprehensive review of the clinical manifestation, diagnostic methods, benign-malignant differentiation, treatment strategies, and prognosis of SFTP. In our case, complete resection and favorable outcomes were achieved through a combination of preoperative interventional embolization and right anterolateral thoracotomy with sternal transection, demonstrating significant potential in treating a giant SFTP.

## Data Availability

The original contributions presented in the study are included in the article/**Supplementary Material**. Further inquiries can be directed to the corresponding author.
