# Supplementary material for: Case report: A case of giant malignant solitary fibrous tumor of the pleura with Doege-Potter’s syndrome and review of the literature
Source: Front Oncol. 2024 Nov 29;14:1437535. doi: 10.3389/fonc.2024.1437535 (PMC11638045; doi:10.3389/fonc.2024.1437535)
Supplement: Supplementary file 1 [file DataSheet1.docx]

**
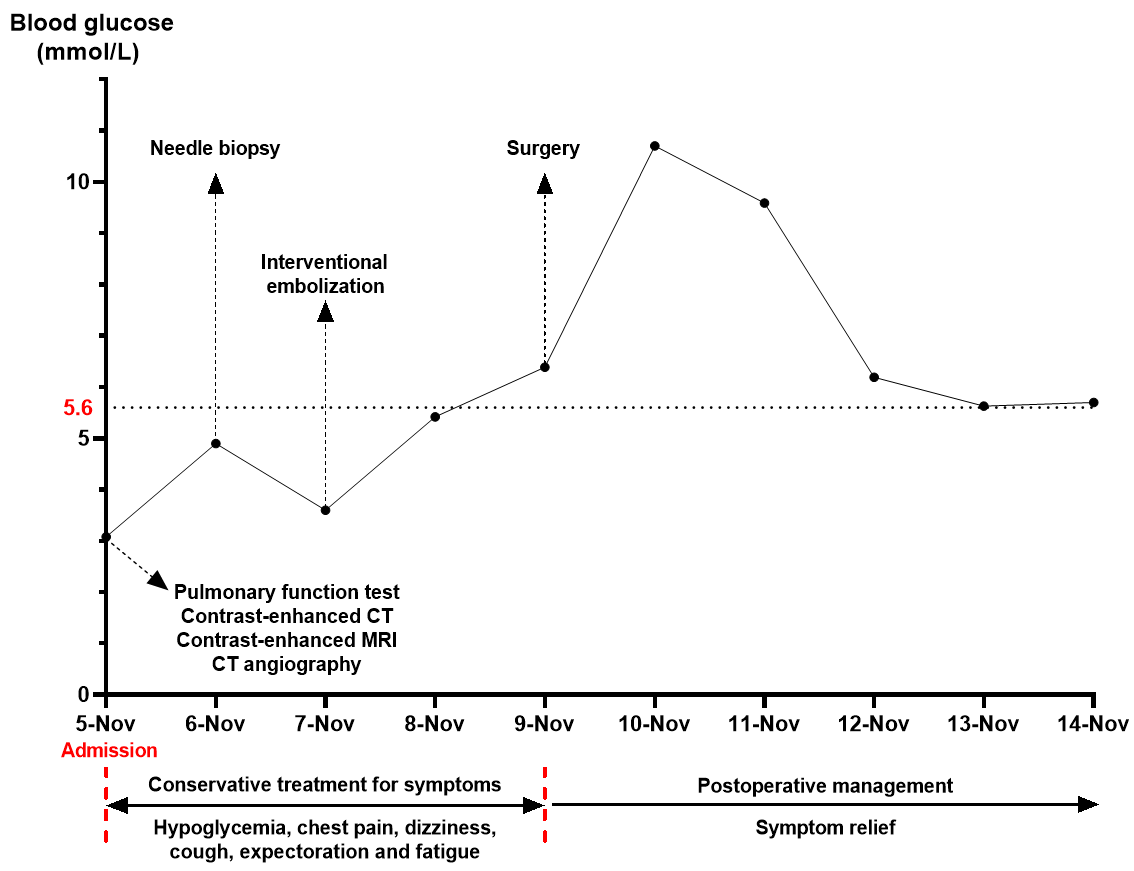
**

**eFigure 1.** The changes of perioperative blood glucose levels and timeline of clinical course.

CT, computed tomography; MRI, magnetic resonance imaging.

**eTable 1:** The summary of the clinical aspects of SFTP

| **Clinical aspects** |  |
| --- | --- |
| **Manifestation** | **Common:** Chest pain, cough, breathlessness, weakness, weight loss……  **Rare:** Doege-Potter’s syndrome, Pierre-Marie-Bamberg syndrome…… |
| **Diagnosis** |  |
| **Radiology** | X-ray, CT, MRI, PET, PET/CT…… |
| **Histopathology** | **Benign SFTP:** Predominantly spindle tumor cells with abundant collagen deposition  **Malignant SFTP:** Fewer collagen bundles, presents hemorrhage, varying degrees of fibrosis, and a predominance of spindle cells with pleomorphic, hyperchromatic and anaplastic nuclei  **Immunopositive:** CD34, CD99, STAT6, Bcl-2, Ki-67, Vimentin……  **Immunonegative:** S-100, keratin, SMA, EMA, CDK4, MDM2,CEA…… |
| **Gene detection** | NAB2-STAT6 fusion, p53 mutation, TERT promoter mutation, BBS9-BRAF fusion…… |
| **Benign-malignant differentiation** |  |
| **Criteria** | The most widely used criteria is proposed by England et al. New scoring systems with other objective parameters have been reported in recent years |
| **Gene expression** | p53 mutation, BBS9-BRAF fusion…… |
| **Radiological features** | CT or MRI features that reveal infiltrating growths, hemorrhages, necrosis, and metastasis |
| **Treatment** | Complete resection **(mainstay)**, radiotherapy, chemotherapy…… |
| **Prognosis** | **Benign pedunculated SFTP:** Satisfied, about 97.5% (10-year OS)  **Local recurrence or metastasis:**  Poor, about 68% (5-year OS) |

SFTP, solitary fibrous tumor of the pleura; CT, computed tomography; MRI, magnetic resonance imaging; PET, positron emission tomography; STAT6, signal transducer and activator of transcription 6; Bcl-2, B-cell lymphoma 2 B-cell lymphoma 2; SMA, smooth muscle actin; EMA, epithelial membrane antigen; CDK4, cyclin-dependent kinase 4; MDM2, mouse double minute 2 homolog; CEA, carcinoembryonic antigen; OS, overall survival
